# Bioinformatics Analysis Reveals the Evolutionary Characteristics of the *Phoebe bournei ARF* Gene Family and Its Expression Patterns in Stress Adaptation

**DOI:** 10.3390/ijms26083701

**Published:** 2025-04-14

**Authors:** Kehui Zheng, Yizhuo Feng, Ronglin Liu, Yanlin Zhang, Dunjin Fan, Kai Zhong, Xinghao Tang, Qinghua Zhang, Shijiang Cao

**Affiliations:** 1 College of Computer and Information Sciences, Fujian Agriculture and Forestry University, Fuzhou 350002, China; zhkehui@fafu.edu.cn; 2 College of Forestry, Fujian Agriculture and Forestry University, Fuzhou 350002, China; 15532106788@163.com (Y.F.); d1315068727@163.com (R.L.); fandunjin@foxmail.com (D.F.); txh060404009@163.com (X.T.); 3 College of Jun Cao Science and Ecology (College of Carbon Neutrality), Fujian Agriculture and Forestry University, Fuzhou 350002, China; 17770899282@163.com (Y.Z.); kaichung2024@163.com (K.Z.); 4 Fujian Academy of Forestry Sciences, Fuzhou 350012, China

**Keywords:** ARF, stress response, *Phoebe bournei*, gene family, expansion

## Abstract

Auxin response factors (ARFs) are pivotal transcription factors that regulate plant growth, development, and stress responses. Yet, the genomic characteristics and functions of ARFs in *Phoebe bournei* remain undefined. In this study, 25 *PbARF* genes were identified for the first time across the entire genome of *P. bournei*. Phylogenetic analysis categorized these genes into five subfamilies, with members of each subfamily displaying similar conserved motifs and gene structures. Notably, Classes III and V contained the largest number of members. Collinearity analysis suggested that segmental duplication events were the primary drivers of *PbARF* gene family expansion. Structural analysis revealed that all *PbARF* genes possess a conserved B3 binding domain and an auxin response element, while additional motifs varied among different classes. Promoter cis-acting element analysis revealed that *PbARF* genes are extensively involved in hormonal responses—particularly to abscisic acid and jasmonic acid and abiotic stresses—as well as abiotic stresses, including heat, drought, light, and dark. Tissue-specific expression analysis showed that *PbARF25*, *PbARF23*, *PbARF19*, *PbARF22*, and *PbARF20* genes (class III), and *PbARF18* and *PbARF11* genes (class V) consistently exhibited high expression levels in the five tissues. In addition, five representative *PbARF* genes were analyzed using qRT-PCR. The results demonstrated significant differences in the expression of *PbARF* genes under various abiotic stress conditions (drought, salt stress, light, and dark), indicating their important roles in stress response. This study laid a foundation for elucidating the molecular evolution mechanism of *ARF* genes in *P. bournei* and for determining the candidate genes for stress-resistance breeding.

## 1. Introduction

Plant developmental trajectories—from syncytium-mediated seed germination to reproductive maturation—are orchestrated through dynamic interactions between intrinsic genetic programs and extrinsic environmental signals. Throughout these processes, transcription factors (TFs) function as molecular rheostats, integrating hormonal and stress signals to regulate growth-defense trade-offs. For instance, Teosinte branched1/Cycloidea/Proliferating (TCP) proteins regulate leaf morphogenesis through cytokinin signaling [1], PIN-FORMED (PIN) auxin transporters direct polar auxin redistribution to coordinate tropic responses [2]; NAM, ATAF1/2, CUC1/2 (NAC), and GATA TFs govern drought tolerance and chloroplast development, respectively [3,4]. Among these regulators, the Auxin Response Factor (ARF) family stands out as a central coordinator of auxin-mediated plasticity, enabling developmental progression with environmental adaptation.

The ARF family represents one of the three core protein families involved in the auxin signaling mechanism, alongside F-box transport inhibitor response 1/auxin signaling f-box protein (TIR1/AFB) auxin co-receptors and Auxin/INDOLE-3-ACETIC ACID (Aux/IAA) transcriptional repressors [5]. As key regulators in the auxin signaling pathway, typical ARFs possess three conserved domains: an N-terminal B3-like DNA binding domain (DBD), a C-terminal dimerization domain (CTD), and a middle region (MR) [6]. The CTD contains two conserved motifs that facilitate both homo- and hetero-dimerization among ARF and Aux/IAA family members [7]. Interaction between ARFs and AUX/IAA proteins occurs through their highly conserved domain III/IV, which plays crucial and coordinated roles in the auxin signaling pathway [8]. At low auxin concentrations, Aux/IAA binds to ARFs and recruits inhibitory proteins, suppressing the expression of auxin-induced genes, thereby inhibiting the downstream signaling pathway. In contrast, elevated auxin levels promote Aux/IAA degradation via the ubiquitin–proteasome pathway, thereby releasing ARFs to activate downstream gene expression [9]. The *ARF* gene family, consisting of transcription factors present in eukaryotes—including animals, plants, and fungi—plays a pivotal role in regulating various biological processes such as the cell cycle, cell proliferation, cell differentiation, and plant responses to environmental stimuli. The distribution of the *ARF* gene family in the plant kingdom has made significant progress [10]. In *Arabidopsis thaliana*, 23 *ARF* genes have been identified in the whole genome, distributed across five chromosomes, providing a foundation for subsequent functional studies [11,12]. In gramineous crops, genome sequencing has revealed 25 *ARF* members in rice (*Oryza sativa*), distributed on 10 of its 12 chromosomes [7,13], and 35 *ARFs* in maize (*Zea mays*) identified through a hidden Markov model (HMM) targeting *ARF* special domains [14,15]. In woody plants, 39 ARFs were found in the genome of *Populus tric*h*ocarpa* [16], and 24 ARFs were found in *Sorg*h*um vulgare* [17], which revealed the expansion difference of the ARF family between woody and herbaceous plants. It is worth noting that although 21 ARF genes have been identified in the genome of the Solanaceae plant *Solanum lycopersicum* [18,19], only six members (*SlARF2/3/4/6/7/8*) have been functionally validated to share conserved roles with *Arabidopsis* homologous genes [20,21,22,23]. In addition, *ARF* genes have also been characterized in other plants, including *Vitis vinifera* [24] and Orchidaceae species [25], underscoring both their evolutionary conservation and functional diversity. However, there are still two major limitations in the existing research. First, *P. bournei* is a camphor tree with unique ecological adaptability, and its *ARF* gene family has not yet completed genome-wide identification, which leads to the lag of auxin regulatory network analysis in this species. Second, although ARF proteins have been confirmed to be involved in key processes such as organ development and stress response [26], more than 60% of the identified family members (such as rice *OsARF9-15*, tomato *SlARF5/9-21*, etc.) still lack systematic functional characterization, which restricts the comprehensive analysis of the regulatory mechanism of this family.

*Phoebe bournei*, a member of the Lauraceae family, is a towering evergreen tree thriving in the tropical and subtropical regions of Fujian, Guangdong, Guangxi, Guizhou, Hubei, Hunan, Jiangxi, and Zhejiang in China [11]. Renowned for its distinct fragrance and excellent resistance to decay, *P. bournei* has been widely used for centuries as a high-quality material for furniture and construction due to its distinctive fragrance and excellent antiseptic properties. Simultaneously, a range of biotechnologies involving exogenous plant hormones is actively being employed to surmount environmental and genetic constraints, improve crop quality, and optimize storage conditions [14,16]. Understanding plant hormones, particularly auxin, plays a crucial role in understanding the functions of various plants, with a particular focus on *P. bournei*, which is the central subject of the experiments delineated herein. In this study, bioinformatics methods were used to analyze and identify members of the *PbARF* family at the whole-genome level. In this study, we aimed to characterize the *PbARF* gene by examining its fundamental physicochemical properties, chromosomal localization, phylogenetic relationships, gene structure, cis-regulatory elements, and expression profiles. Delving into the *PbARF* family holds promise for a deeper understanding of its structure and functions and facilitates future experimental research.

## 2. Results

### 2.1. Identification and Phylogenetic Analysis of ARF Gene Family in Phoebe bournei

The conserved domains of the ARF1 gene family in *A. thaliana* were downloaded from PlantTFDB, and the conserved domains between *P. bournei* and *A. thaliana* were compared by local BLASTp search. Combined with NCBI BLASTp and HMMER tools and the ARF1 HMM model (PF06507) in the Pfam database, we identified and verified the candidate ARF1 gene in *P. bournei*. As a result, 25 *PbARF* family members were identified and named *PbARF1* to *PbARF25* (Table 1). The number of amino acids in the 25 *ARF* genes ranged from 490 to 1444, with *PbARF9* having the most and PbARF4 having the fewest. The corresponding molecular weights of the proteins varied from 35,145.69 (*PbARF21*) to 165,759.74 Da (*PbARF9*). The isoelectric points ranged from 5.57 to 9.27; they were greater than 7 for *PbARF3*, *PbARF4*, *PbARF7*, *PbARF8*, *PbARF13*, *PbARF14*, and *PbARF24* and less than 7 for the rest, indicating that there are both acidic and basic proteins in this family. The instability indices ranged from 43.97 (*PbARF3*) to 71.90 (*PbARF23*). The lipolysis index ranged from 64.68 (*PbARF18*) to 88.18 (*PbARF13*). The average coefficients of hydrophilicity ranged from −0.620 (*PbARF18*) to −0.221 (*PbARF13*), indicating that the *PbARF* proteins have high hydrophilicity. The prediction results of subcellular localization showed that *PbARF13* was located in the chloroplast, *PbARF10* and *PbARF17* were located in the peroxisome, and the remaining PbARF genes were located in the nucleus.

**Figure 1:**
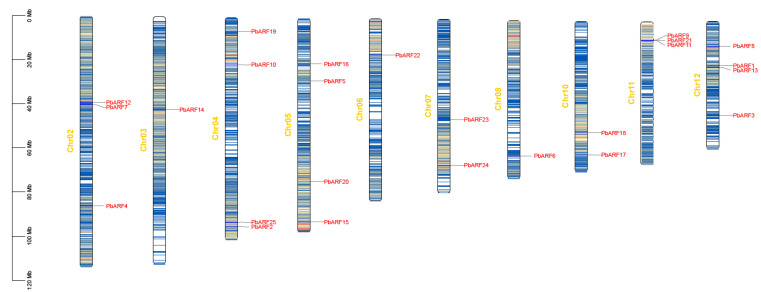
Chromosomal distribution map of *ARF* genes in *Phoebe bournei*. The left side of each bar shows the chromosome number, and the left scale of the bars indicates the relative length of a chromosome. The left scale unit is Mb, and the short line shows the approximate location of the *PbARF* gene on the corresponding chromosome.

In this study, a total of 25 *ARF* genes were identified in *P. bournei* and mapped to their locations on chromosomes (Figure 1). The results showed that the distribution of *PbARF* genes was uneven across different chromosomes. The chromosomes with the highest number of *PbARF* genes each contained four *PbARF* genes: Chr12 (*PbARF1*, *PbARF3*, *PbARF8*, and *PbARF13*), Chr05 (*PbARF5*, *PbARF15*, *PbARF16*, and *PbARF20*), and Chr04 (*PbARF2*, *PbARF10*, *PbARF19*, and *PbARF25*). Chromosomes Chr03 (*PbARF14*), Chr06 (*PbARF22*), and Chr08 (*PbARF6*) had only a single gene, which was characterized by only one *PbARF* gene. Both chromosomes Chr02 (*PbARF4*, *PbARF7*, and *PbARF12*) and Chr11 (*PbARF9*, *PbAR11*, and *PbARF21*) each contained three *PbARF* genes. Notably, the genes *PbARF9*, *PbAR11*, and *PbARF21* on Chr11 were closely clustered together. In contrast, Chr07 (*PbARF23* and *PbARF24*) and Chr 10 (*PbARF17* and *PbARF18*) each contained only two *PbARF* genes.

**Figure 2:**
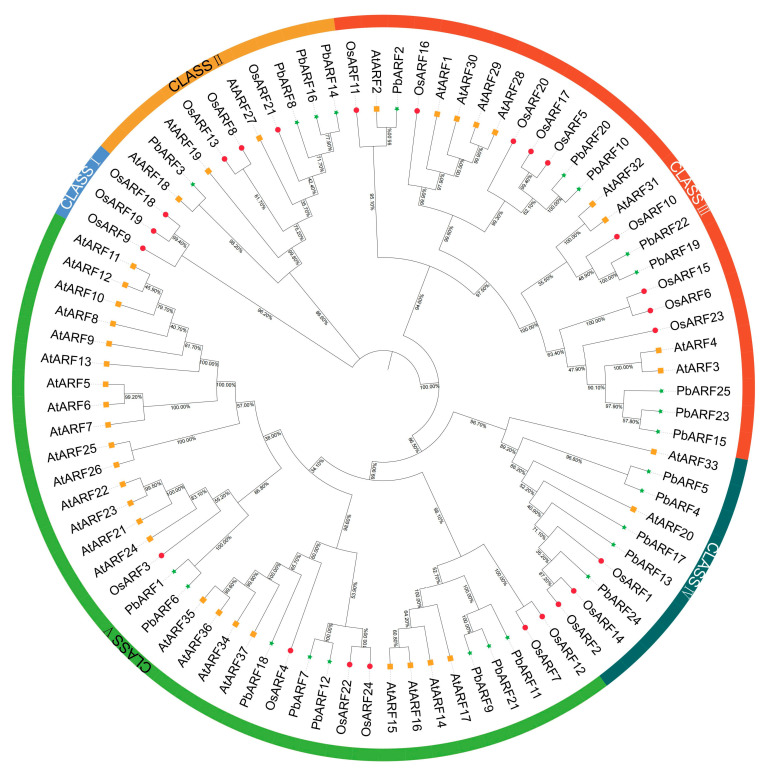
Phylogenetic tree of 86 ARF genes from *Arabidopsis thaliana* (At) and *Oryza sativa* (Os) and *Phoebe bournei* (Pb). The phylogenetic tree was constructed using the neighbor-joining method with MEGA11.0. All ARFs were categorized into five classes, each class represented by a different color: CLASS I is shown in blue, CLASS II in yellow, CLASS III in red, CLASS IV in dark green, and CLASS V in light green.

To further investigate the functional diversification and evolutionary relationships of the ARF family, a phylogenetic tree was constructed using the neighbor-joining (NJ) method based on ARF protein sequences obtained from *Arabidopsis thaliana*, *Oryza sativa*, and *P*. *bournei* (Figure 2). This analysis utilized 25 *PbARF* protein sequences and other 61 *ARF* protein sequences from *Arabidopsis* and rice. A total of 86 *ARF* transcription factor protein sequences from various plants were categorized into five classes, ranging from I to V. Class III and V contain the largest number of *PbARF* proteins, together accounting for 64% of all *PbARFs*. Class IV contains five *PbARF* proteins; CLASS II has four. Notably, CLASS I was devoid of any *PbARF* proteins contained three *OsARF* proteins (*OsARF9*, *OsARF18*, and *OsARF19*). Since the genes within each taxonomic group are derived from different species, it indicates that these genes have a homologous relationship among different species. For example, in CLASS I, there are genes from Os, Pb, Pt, and At, which suggests that these genes have a similar evolutionary history in these species. The bootstrap values associated with the nodes of the phylogenetic tree were generally above 50, indicating moderate to high confidence in the inferred evolutionary relationships.

### 2.2. Protein Motif and Gene Structure Analysis of PbARF Genes

**Figure 3:**
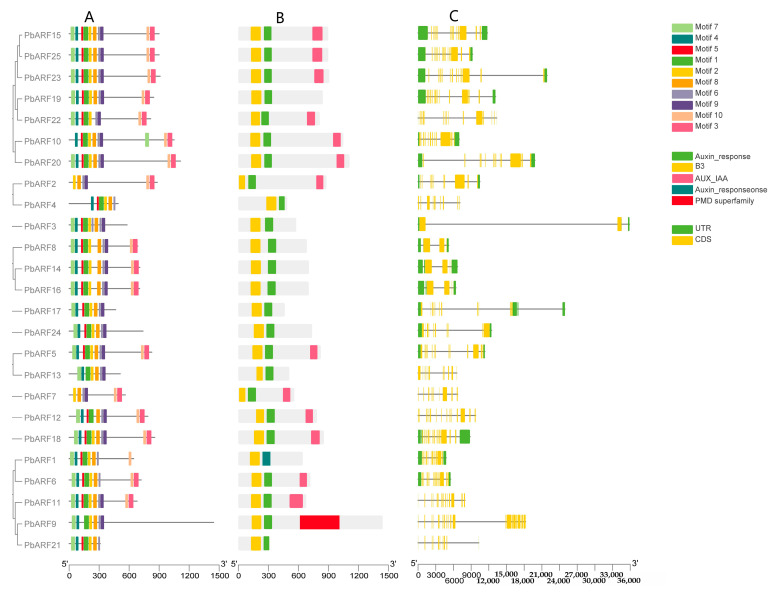
Schematic diagram of *PbARF* conserved motifs and gene structure. (**A**) Motifs in *PbARF* proteins, from motif 1 to motif 10, are marked by different colors. Black lines indicate non-conserved protein sequences. (**B**) Conserved domains of *PbARF* genes are depicted in different colors. (**C**) Gene structure of *PbARFs*. The yellow boxes represent coding sequences (CDS), and the green boxes indicate untranslated regions (UTRs) of *PbARFs*. The relative positions are consistently represented according to the kilobase scale at the bottom of the figures.

To explore the structural diversity of the *PbARF* gene family, we analyzed the conserved motifs and gene structures of *PbARF* genes. Using 25 *ARF* amino acid sequences, we predicted the conserved motifs within these genes (Figure 3). Motif analysis indicated that all *PbARF* proteins contain Motifs 2 and 8, indicating a high level of conservation of these motifs across the *PbARF* gene family. Within CLASS III, Members *PbARF15*, *PbARF25*, *PbARF23*, *PbARF19*, *PbARF22*, and *PbARF20* exhibited an identical motif composition (Motifs 1 to 10), suggesting a high level of structural homogeneity within this class. In contrast to Class IV *PbARF* genes, those in Class III and Class V possess additional motifs, specifically Motif 3 and Motif 10, indicating intrinsic variability among the different classes of *PbARF* genes. Regarding conserved domains, all *PbARF* genes include a B3 domain and an auxin response element. Notably, several genes in Class III (*PbARF15*, *PbARF25*, *PbARF23*, *PbARF22*, *PbARF10*, *PbARF2*) and Class V (*PbARF7*, *PbARF12*, *PbARF18*, *PbARF6*, *PbARF11*) also harbor an Aux/IAA domain. Furthermore, the *PbARF9* gene contains a PMD superfamily domain. Except for *PbARF3*, which has only two exons, all other *PbARF* genes exhibit a higher number of exons, underscoring the diversity and complexity in the transcriptional profiles among the *PbARF* genes.

### 2.3. Cis-Acting Element Analysis of PbARF Gene Family

**Figure 4:**
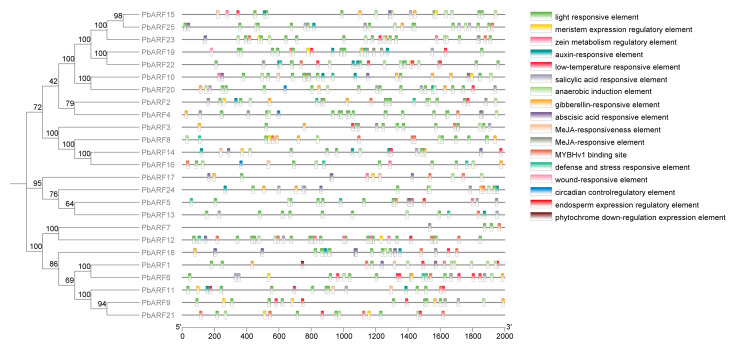
Schematic representation of important cis-acting elements in the promoter regions of *PbARF* genes, correlated with the phylogenetic classification of their encoded *PbARF* proteins. Different types of promoters are shown in different colors.

Cis-acting elements are DNA sequences located in the promoter region upstream of genes, where they serve as binding sites for transcription factors to regulate gene expression. In our investigation of the *PbARF* gene function, we predicted the cis-acting elements. As illustrated in Figure 4, a total of 17 distinct cis-acting elements were identified within the *PbARF* gene. These elements include those responsive to various hormones, such as auxin, gibberellin (GA), salicylic acid (SA), and methyl jasmonate (MeJA), suggesting a significant role for the *PbARF* gene in hormonal responses. For instance, the promoter regions of *PbARF1* and *PbARF9* contain a relatively large number of auxin-responsive elements, suggesting their putative central roles in the auxin signaling cascade. Similarly, *PbARF10* and *PbARF20* promoters are enriched with low-temperature responsive elements and salicylic acid-responsive elements. This may be related to their functions in plants’ response to environmental stresses. The promoter regions of *PbARF3* and *PbARF8* contain a relatively large number of gibberellin-responsive elements and abscisic acid-responsive elements. This may be associated with their roles in plant hormone regulation.

### 2.4. Synteny Analysis of PbARF Gene Family

**Figure 5:**
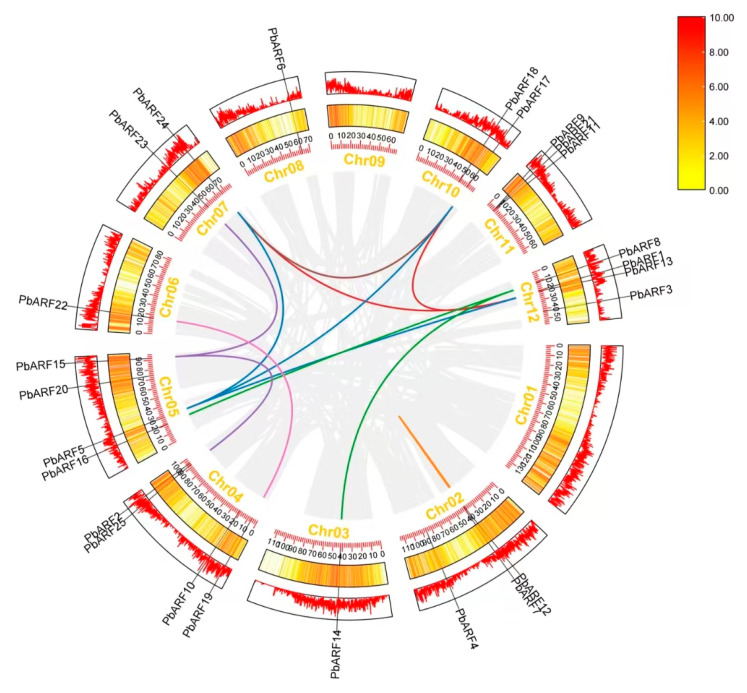
Intraspecific synteny of the *ARF* gene family in *Phoebe bournei*. The two outer rings represent the gene density per chromosome, and the grey line represents the synteny block in the genome. Lines of the same color represent duplicated *PbARF* gene pairs, and the chromosome number is shown in a rectangular box for each chromosome.

**Figure 6:**
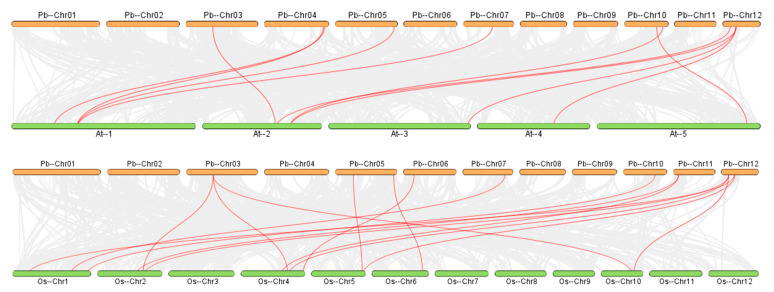
Interspecific *s*ynteny analysis of *ARF* genes in *Arabidopsis*, rice, and *Phoebe bournei*. Gray lines indicate covariance between the genomes of *Phoebe bournei* and other plants, and red lines indicate covariance of the *PbARF* genes with ARF genes in other plants.

To explore the evolutionary relationships of *PbARF* genes, we performed interspecies covariance and intraspecies covariance analyses (Figure 5 and Figure 6). The intraspecific covariance analysis revealed one tandem duplication event (*PbARF7* and *PbARF12*) and 11 segmental duplication events (*PbARF22* and *PbARF19*, *PbARF23* and *PbARF15*, *PbARF15* and *PbARF2*, *PbARF24* and *PbARF5*, *PbARF5* and *PbARF13*, *PbARF5* and *PbARF17*, *PbARF16* and *PbARF8*, *PbARF8* and *PbARF14*, *PbARF13* and *PbARF17*, *PbARF13* and *PbARF24*, *PbARF24* and *PbARF17*) in the evolution of the *P*. *bournei* genes. The *PbARF* gene produced by non-replication events in Figure 5 may be derived from a variety of mechanisms, including gene loss, independent origin, and transposition events. According to our analysis, some *PbARF* genes may have lost their homologous genes during evolution, thus forming a unique function; other genes may appear independently in the process of evolution without obvious replication events. Some *PbARF* genes may move in the genome through the transposition mechanism, thus forming a new locus. These findings indicate that the *PbARF* transcription factor family is primarily composed of genes derived from high-segment (large-scale) duplication events, with additional genes originating from low-tandem (small-scale) duplication events. The distribution of these duplicated genes indicates that there may be functional or evolutionary connections among the *PbARF* genes located on different chromosomes. In a comprehensive comparison of homologous gene pairs in the genomes of *A. thaliana*, *O. sativa*, and *P. bournei*, we identified 11 pairs of collinear gene pairs between the *PbARF* gene of *P. bournei* and the *AtARF* gene of *A. thaliana*, and 15 pairs of collinear gene pairs between the *PbARF* gene and the *OsARF* gene of rice. These findings reveal that these species are highly conserved in genome arrangement and evolutionary relationships, indicating that the *ARF* gene family maintains significant collinearity despite evolutionary divergence. The conservation of gene order and function in these genomes provides valuable insights into the molecular mechanisms of plant development and stress adaptation. It also lays a foundation for predicting the function of *PbARF* genes based on homologous genes that have been fully studied in model species. This analysis not only reinforces the importance of collinearity studies in elucidating the evolution of gene families but also highlights the need for further research on the specific roles of these conserved genes in different plant species. By using these collinear data, future functional studies can explore how conserved *ARF* genes contribute to the unique phenotypic and ecological adaptation of *P*. *bournei* and other plants.

### 2.5. Expression Analysis of PbARF Gene in Different Tissues of Phoebe bournei

**Figure 7:**
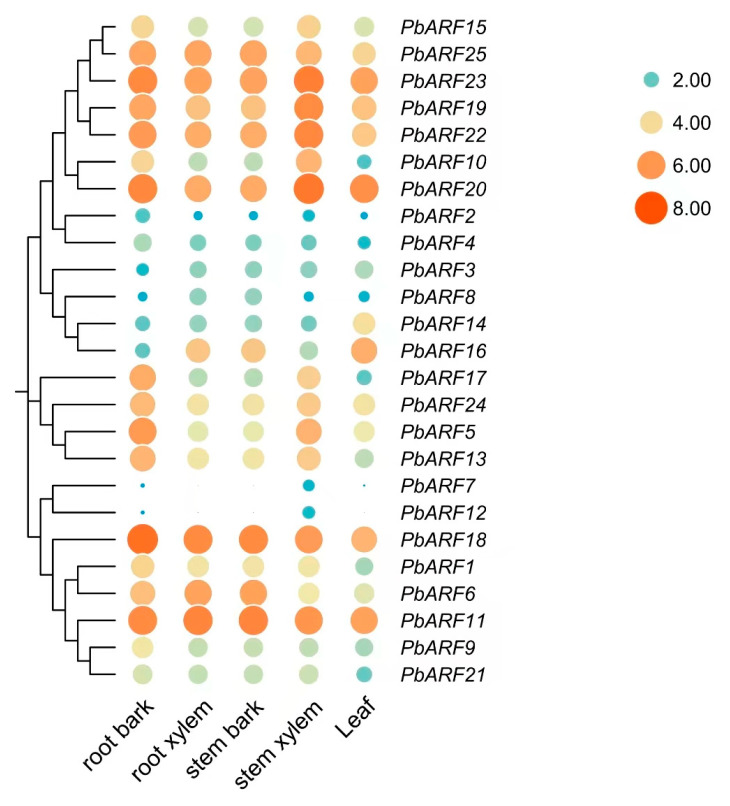
Tissue-specific gene expression patterns of 25 *PbARF* genes were analyzed across root bark, root xylem, stem bark, stem xylem, and leaf tissues. Red indicates high abundance (higher FPKM value), blue indicates low abundance (lower FPKM value), and the size of each point increases with the value of high abundance.

To explore the functional role of *PbARF* genes in *P. bournei* tissues, we analyzed the expression profiles of 25 *PbARF* genes across different tissues. As shown in the heatmap (Figure 7), *PbARF* genes exhibited higher expression in root bark, root xylem, stem bark, and stem xylem compared to leaves. Notable variation in expression levels was observed among *PbARF* genes from different classes. In particular, Class III shows significantly higher expression across all five tissues when compared to genes from other classes. Specifically, *PbARF25*, *PbARF23*, *PbARF19*, *PbARF22*, and *PbARF20* (Class III) along with *PbARF18* and *PbARF11* from Class V consistently exhibit high levels of expression. In contrast, genes such as *PbARF2*, *PbARF3*, *PbARF4*, *PbARF7*, and *PbARF12*, which belong to Classes II, IV, and V, exhibit relatively lower expression across the tissues. These results suggest that genes from different classes have differential expression across tissues, with some consistently showing lower expression within each class.

### 2.6. Expression Analysis of the PbARF Gene Under Different Treatments

**Figure 8:**
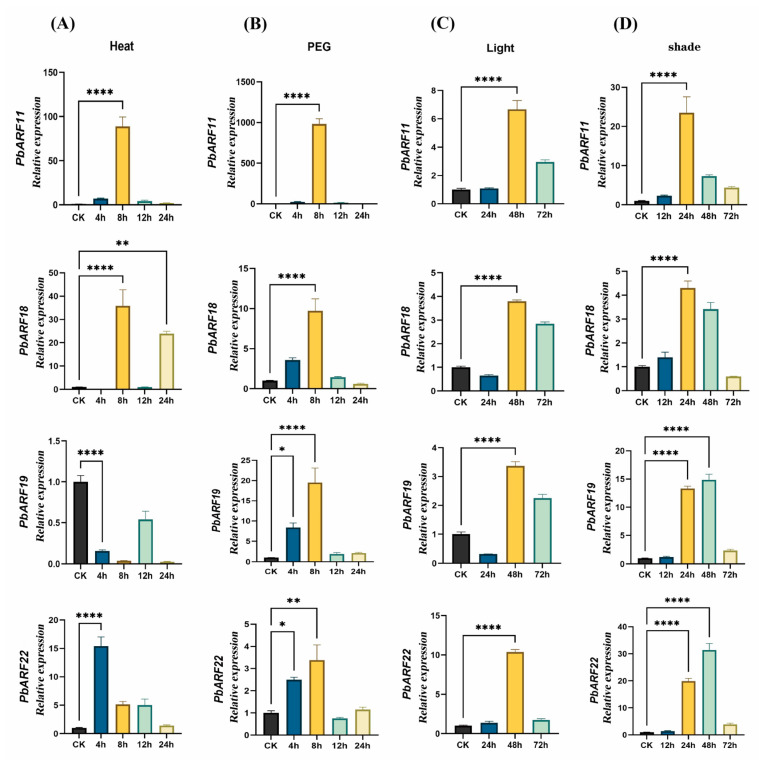
The expression profiles of the five selected *PbARF* genes in *Phoebe bournei* were detected by qRT-PCR under heat, PEG, light, and darkness conditions. (**A**) Relative gene expression levels under heat treatment over periods of 4, 8, 12, and 24 h. (**B**) Relative gene expression levels under drought stress over the same periods (4, 8, 12, and 24 h). (**C**) Relative gene expression levels under treatment of light conditions over the same periods (4, 8, 12, and 24 h). (**D**) Relative gene expression levels under conditions without light (* *p* < 0.05, ** *p* < 0.01, and **** *p* < 0.0001).

Five typical genes, *PbARF11*, *PbARF18*, *PbARF19*, *PbARF22*, and *PbARF25*, were chosen to examine their expression under heat, drought, light, and dark conditions. The results demonstrate that *PbARF* gene expression is influenced by high temperature, drought, and variations in light intensity (Figure 8). Under heat stress, *PbARF11*, *PbARF22*, and *PbARF18* exhibited significantly increased expression at 8 h post-treatment, indicating their potential involvement in the heat stress response. During polyethylene glycol (PEG)-induced simulated osmotic stress, *PbARF18*, *PbARF19*, and *PbARF22* displayed significant upregulation in gene expression from 0 to 8 h, while *PbARF11* showed particularly high expression levels at the 8-h mark, and PbRAF25 was downregulated at 4 h and slightly upregulated at 8 h. These findings imply that *PbARF11*, *PbARF18*, *PbARF19*, and *PbARF22* may play crucial roles in drought stress adaptation. In response to light exposure, *PbARF11* and *PbARF22* showed upregulated expression between 0 and 48 h, whereas *PbARF18* and *PbARF19* exhibited a significant increase in expression relative to baseline levels, and PbARF25 was significantly downregulated at 0–48 h. These results suggest that *PbARF11*, *PbARF18*, *PbARF19*, *PbARF22*, and *PbARF25* may play a key role in light stress adaptation. Under shade conditions, the selected *PbARF* genes, including *PbARF11*, *PbARF18*, *PbARF19*, and *PbARF22*, exhibited enhanced expression levels from 0 to 24 h, with a notable increase compared to light treatment conditions. Collectively, these observations indicate that *PbARF* gene expression is differentially regulated in response to light and dark environments.

## 3. Discussion

Throughout their life cycle, plants are exposed to a variety of biotic and abiotic stresses that can affect their normal growth and reproduction [27]. To cope with these stresses, plants have evolved a complex network of signaling pathways involving multiple regulators, which enable responses to environmental stresses such as salinity, high and low temperatures, drought, pests, and diseases [28,29]. Phytohormones, such as abscisic acid, ethylene, salicylic acid, and jasmonic acid, play key roles in regulating plant response to adversity [30,31]. Auxin, the first discovered and most widely studied phytohormone, regulates plant growth and stress-responsive processes through the modulation of *ARF* genes [9].

*ARFs* are a family of genes encoding transcription factors that play a crucial role in plant growth and developmental processes [32]. To better elucidate the function of *P. bournei ARFs* in stress responses, this study focuses on the main structural characteristics of the *P. bournei ARF* gene family. In this study, we searched for ARF genes in the *P. bournei* genome and identified 25 members. Genome doubling and diversification have played important roles in the evolution of the *ARF* gene family [33]. The number of *ARF* genes in *P. bournei* is comparable to that in other plants, such as *Arabidopsis* (23), rice (25), *Solanum lycopersicum* (21), and *Vitis vinifera* (19), suggesting that the *PbARF* gene family is relatively conserved and has not undergone large-scale gene amplification as observed in rice and *Arabidopsis* during the evolutionary process [11,13,14,34,35]. Genome-wide analysis indicated that 25 *PbARFs* and 61 *ARFs* from two other plant species were categorized into five classes. CLASS III and CLASS V *PbARF* genes contained the most *PbARF* genes, while CLASS I lacked ARF genes and contained only three *OsARF* genes, which may reflect that *P. bournei*, as a woody plant, adapted to specific environmental pressures (such as high temperature and drought) through gene duplication events (such as fragment replication) during evolution. This finding contrasts with the evolutionary patterns of tomato *SlARF* and poplar *PtrARF*, highlighting the uniqueness of the woody plant gene family expansion [26,36].

The number and characteristics of the domains in the ARF protein sequences provide valuable insights for predicting their functions in *P. bournei*. Typical ARF proteins possess three conserved regions: a DNA-binding B3 domain, a variable middle region domain that modulates gene expression through activation or repression, and a C-terminal domain that enables protein-protein interactions via dimerization, thereby precisely controlling auxin signaling pathways [37,38]. In this study, all *PbARF* genes contain a B3 domain and an auxin response domain, similar to *Arabidopsis ARF* genes [39]. *PbARF* genes in CLASS III and CLASS V have additional AUX/IAA conserved domains not found in other classes, indicating that these two classes are more conserved compared to the others. The *ARFs* within the same class exhibit similar intron-exon structures, suggesting structural conservation within classes. However, nearly all CLASS II members have lost two motifs, probably due to selective pressure during evolution.

In this study, a large number of regulatory motifs related to hormone response, stress signal transduction, light response, and growth and development were found by analyzing the cis-acting elements in the promoter region of the *PbARF* gene. The presence of these motifs means that *PbARF* genes are integrated into a complex regulatory network that modulates plants response to environmental cues and also shows that we can optimize plant growth and development or improve crop quality by regulating ARFs. Additionally, the cis-element prediction revealed a substantial presence of light-responsive elements across all the PbARF gene classes, implying a crucial role for these genes in light response processes. For example, the distributions of light-responsive elements and meristem expression regulatory elements in the promoter regions of *PbARF15* and *PbARF25* are quite similar, which may suggest that these genes share similar functions or regulatory mechanisms during the evolutionary process.

During the evolution of gene families, gene duplications—such as whole genome duplication (WGD), tandem duplication (TD), chromosomal segmental duplication (SD), and retrotransposition (TRD)—are key driving forces [40]. Among these mechanisms, tandem duplication and segmental duplications are the primary mechanisms for the formation and expansion of gene families [41,42]. Gene loss and amplification events have led to structural divergence in gene families between herbaceous and woody plants [43,44]. In rice, segment duplication has driven the expansion of the *ARF* gene family [45,46]. Similarly, extensive segmental duplication in the *PbARF* gene family has led to an increase in the number of *PbARF* genes. Between *P. bournei* and *Arabidopsis*, 11 pairs of orthologous genes were identified, while 15 pairs were found between *P. bournei* and rice. These findings suggest that the *ARF* genes of *P. bournei* may be more closely related to the *ARF* genes of rice, with significant conservation observed in the genomic arrangement and evolutionary relationships between the two species. These results stand in sharp contrast to the expected evolutionary divergence between monocotyledons and dicotyledons. This could be attributed to a certain number of *ARF* gene losses that occurred during the evolutionary process of *P. bournei’s ARF* gene family.

Expression patterns of *PbARF* genes were investigated in five different tissues (Figure 8). Several *PbARF* genes displayed tissue-specific expression profiles in *P. bournei*. In *Arabidopsis*, *ARF7* affects phototropism in the hypocotyl and the formation of lateral roots, while *ARF6* and *ARF8* exhibit functional redundancy in regulating hypocotyl elongation and xylem development [47,48,49,50]. In our analysis, *PbARF11* and *PbARF18*, which belong to the same class as *AtARF7*, have higher gene expression in root xylem tissue. This indicates that *PbARF11* and *PbARF18* may be involved in regulating the differential growth of roots through a similar mechanism to *AtARF7*, indicating that these genes may be functionally conserved in root development and light response. Previous studies have shown that the *ARF* gene family plays an important role in stress responses, including heat and drought, by regulating the expression of stress-responsive genes and modulating plant growth and development under heat stress. For example, in tomato plants (*Solanum lycopersicum*), drought stress significantly upregulates the expression of *SlARF1*, *SlARF4*, *SlARF6B*, *SlARF10A*, and *SlARF18*, and leads to a noticeable upregulation of the expression of three *TgARF* genes: *TgARF1*, *TgARF2*, and *TgARF8* [36,51]. Consistent with previous studies, our study found that *PbARF11*, *PbARF18*, *PbARF19*, and *PbARF22* were significantly upregulated under drought stress, indicating that these genes may play an important role in the response of plants to water stress and may be involved in the regulation mechanism of plant stress resistance. Among them, the significant upregulation of *PbARF18* under drought stress is similar to the response mechanism of tomato *SlARF18*, but the unique Aux/IAA domain of the *P. bournei* gene may give it an additional regulatory level [36]. Under light and shade conditions, the expression of *PbARF* genes showed different patterns. Some genes were upregulated or significantly increased under light treatment, while others were significantly downregulated. Under shading conditions, the expression levels of most *PbARF* genes increased, with significant differences compared to the light treatment group. These indicate that the *PbARF* gene plays a key role in plant adaptation to light stress and that their expression is regulated by light conditions, reflecting the response mechanism of plants to light signals under different light conditions and the potential role in photoperiod regulation. However, further in-depth research is required to fully understand the function of *ARF* genes in response to abiotic stressors in a wider range of plant species.

This study not only determined and examined the expression of *PbARF* using qRT-PCR but also systematically analyzed the *ARF* gene family of *P. bournei*, highlighting the potential role of *PbARF* in the physiological response of *P. bournei* to heat, salt, and light and shade stresses. This study provides a basis for elucidating the molecular mechanisms of the *P. bournei* ARF response to heat, salt, light, and shade stresses and offers insights for future improvement of *P. bournei* using genetic engineering methods.

## 4. Materials and Methods

### 4.1. Genome Data and Plant Material Source

The genome sequence data and annotation information of *P. bournei* were downloaded from the Sequence Archive of China National GeneBank Database (CNSA) with accession number CNP0002030 (https://db.cngb.org/search/project/CNP0002030/ (accessed on 1 October 2024)) [52]. Genome sequence files of *A. thaliana* and *O. sativa* were acquired from EnsemblPlants (https://plants.ensembl.org/index.html/ (accessed on 1 October 2024)) and Phytozome v13 (https://phytozome-next.jgi.doe.gov/ (accessed on 1 October 2024)), respectively. The RNA-seq data from different tissues of *P. bournei* were downloaded from BioProject with accession number PRJNA628065 (https://ngdc.cncb.ac.cn/bioproject/ (accessed on 1 October 2024)). Plant materials were derived from one-year-old *P. bournei* seedlings cultured in an artificial climate box under different treatments. *P. bournei* seedlings of similar growth potential, aged 1 year, were selected for treatment, and the materials were divided into the control group and a stress treatment group, with 30 plants in the treatment group and 3 plants in the control group. Every 2 plants in the treatment group were used as biological replicates, and 3 groups of biological replicates were set for each period. After various treatments, leaf samples from *P. bournei* were collected and stored in liquid nitrogen at −80 °C for RNA extraction.

### 4.2. Identification and Analysis of Physical and Chemical Properties

In order to identify the members of the *ARF1* gene family in *P.bournei*, we first downloaded the conserved domain of the *ARF1* gene family in *A. thaliana* from PlantTFDB (https://planttfdb.gao-lab.org/ (accessed on 20 October 2024)). Using the local BLASTp search tool, the conserved domains between *P. bournei* and *A. thaliana* were compared to screen the candidate *ARF1* genes in *P. bournei* [53]. Subsequently, we remove the duplicates in the BLASTp search results to ensure the accuracy of the data. To further validate and identify members of the *ARF1* gene family, we downloaded the HMM (PF06507) of the *ARF1* conserved domain from the Pfam database and used HMMER-3.2.1 (https://www.ncbi.nlm.nih.gov/Structure/bwrpsb/bwrpsb.cgi (accessed on 20 October 2024)) to search, setting the expected value to be less than 10^−5^, and other parameters remain default. By comparing the protein sequences obtained by BLASTp and HMMER, we selected the consistent sequences for subsequent analysis. Finally, in order to ensure the accuracy of the identified *ARF1* gene family members, we used the SMART database (https://smart.embl.de/ (accessed on 20 October 2024)), the InterPro database, and the NCBI-CDD database (https://www.ncbi.nlm.nih.gov/Structure/bwrpsb/bwrpsb.cgi (accessed on 20 October 2024)) to further detect the conserved domains of these protein sequences, excluding those that do not contain the ARF1 conserved domain protein sequence. After the identification of *ARF1* genes in *P. bournei*, the online website ExPASy (https://web.expasy.org/protparam/ accessed on 20 October 2024) was used to analyze the physical and chemical properties of the identified ARF1 protein, and the WOLF PSORT website (https://wolfpsort.hgc.jp/ accessed on 20 October 2024) was used for subcellular localization analysis [54].

### 4.3. Chromosomal Distribution and Gene Duplication of PbARF1 Genes

TBtools was used for grepping the chromosomal location information of the *PbARF1* genes from the genome (FASTA) file and the annotation (GFF) file of *P. bournei* [55]. Gene duplication and syntenic relationships of *PbARF1* were determined using MCScanX (https://smart.embl.de/ (accessed on 21 October 2024)) with default parameters and plotted using TBtools-v2.10 [56].

### 4.4. Collinearity Analysis of PbARF1 Genes

The syntenic relationships between PbARF1 genes and ARF1 genes from *A. thaliana* and *O. sativa* were determined by using MCScanX v2.0 software. TBtools-v2.10 was used for visualization.

### 4.5. Phylogenetic Analysis

The sequences of ARF1 proteins of *P. bournei*, *A. thaliana*, and *O. sativa* were aligned using the MUSCLE program of MEGA11 (accessed on 23 October 2024) with default settings. A maximum likelihood phylogenetic tree was constructed using bootstrap replications: 1000 [57]. ITOL (https://itol.embl.de/ (accessed on 17 October 2024)) was used to improve and refine the appearance of the phylogenetic tree.

### 4.6. Analysis of Conserved Motifs and Gene Structures

The protein sequences of *P. bournei* were analyzed using the online software MEME, version 5.5.7, and the predicted value of the motif number was 10. We used the Batch CD search with default parameters to identify the conserved domains in the PbARF1 protein.

### 4.7. Multiple Sequence Alignment and Promoter Cis-Element Analysis of PbARF1 Genes

Multiple sequence alignment of *PbARF1* was performed using Jalview software version 2.11.3. To investigate the cis-acting elements in the sequence, we extracted the upstream 2000 bp sequences from the *P. bournei* genome. The online software PlantCARE (http://bioinformatics.psb.ugent.be/webtools/plantcare/html/ (accessed on 27 October 2024)) was used to analyze the cis-acting regulatory elements in the promoter region of the *PbARF1* genes. After selection and categorization, the data were visualized by TBtools-v2.10.

### 4.8. Different Plant Tissues and Abiotic Stress Treatments

The fragments per kilobase of transcript per million fragments mapped (FPKM) transcriptomic data from five different tissues (leaf, root xylem, stem xylem, root bark, and stem bark) were used to construct an expression profile using TBtools v2.154. The *P. bournei* material used in this experiment is a one-year-old seedling purchased from Fujian Academy of Forestry Sciences. It is cultivated under natural conditions and treated with heat, PEG, light, and dark. According to the previous research in related fields and in order to effectively and systematically capture the dynamic change trend of gene expression at different times, this study selected heat (40 °C) and PEG (10% PEG6000); five gradients were set to 4 h, 8 h, 12 h, 24 h, and the control group (0 h). During the light and dark treatments, the five gradients were set to 0 h, 12 h, 24 h, 48 h, and 72 h, respectively. Each treatment was repeated 3 times, and mature leaves were collected within the specified time. After treatment, the leaves were immediately stored in liquid nitrogen at −80 °C for future RNA extraction.

### 4.9. qRT-PCR Analysis

Total RNA was extracted using the HiPure Plant RNA Mini Kit (Magen, Shanghai, China), and cDNA was synthesized using the Prime Script RT reagent Kit (Perfect Real Time from Takara, Dalian, China). Specific primers were designed in the non-conserved region of the target gene using Primer 3.0 software and synthesized by Fuzhou Qingbaiwang Biotechnology Company (Fuzhou, China). Real-time fluorescence quantitative analysis was performed with the following reaction setup: cDNA template (1 µL), cDNA template SYBR Premix Ex TaqTM II (10 µL), specific primers (2 µL) (Table S1), and ddH2O reaction program (7 µL). The thermal cycling conditions were: 95 °C for 30 s; 95 °C for 5 s; 60 °C for 30 s; 95 °C for 5 s; 60 °C for 60 s; and a final 30 s at 50 °C, with a total of 40 cycles. The internal reference gene used was PbEF1α (GenBank No. KX682032). The expression level of the target gene was calculated using the 2^−∆∆Ct^ method, and the quantitative data were analyzed with a *t*-test using SPSS26 software. Finally, graphs were constructed using GraphPad Prism 9.0 (software website: https://www.graphpad.com/ (accessed on 17 November 2024)).

## 5. Conclusions

The identification and characterization of the *ARF* gene family in *P. bournei* have unveiled the structure of the *PbARF* genes and highlighted their key features related to the plant’s resistance to adverse stress. Most *PbARF* genes contained a significant number of exons, except for PbARF3, which had only two exons. Comparative genomics of *P. bournei* with *Arabidopsis* and rice indicate that these three gene families share a common origin but have undergone independent evolutionary histories. Overall, the gene family structure of *P. bournei* is more conserved with the monocot rice than with the dicot *Arabidopsis*. The expression profiles of *PbARF* genes under various conditions allow us to pinpoint genes with regulated expression patterns. Moreover, the chromosomal arrangement and evolutionary analysis of these genes provide valuable insights into the evolutionary characteristics of the *P. bournei* genome.

## Figures and Tables

**Table 1:** Detailed information on 25 *PbARF* genes in *Phoebe bournei* and their encoded proteins.

Gene Name	Gene ID	AA/aa	MW/kDa	pI	II	AI	GRAVY	Subcelluar Localization	CLASS
*OF09010*	PbARF1	715	80,305.10	6.04	58.31	73.61	−0.470	Nucleus	V
*OF01795*	PbARF2	1059	117,250.14	5.57	57.40	73.98	−0.485	Nucleus	III
*OF13500*	PbARF3	571	62,566.62	7.56	44.58	71.84	−0.337	Nucleus	II
*OF12274*	PbARF4	490	53,867.87	9.27	56.49	81.41	−0.324	Nucleus	IV
*OF00399*	PbARF5	826	91,562.44	6.33	43.97	73.16	−0.434	Nucleus	IV
*OF14335*	PbARF6	720	80,909.46	6.07	57.04	73.50	−0.502	Nucleus	V
*OF02341*	PbARF7	561	62,376.75	8.02	50.82	70.91	−0.466	Nucleus	V
*OF14461*	PbARF8	674	74,422.02	7.01	50.77	71.31	−0.413	Nucleus	II
*OF14278*	PbARF9	1444	165,759.74	6.70	46.97	76.20	−0.467	Nucleus	V
*OF15166*	PbARF10	1057	117,517.64	6.22	61.66	77.31	−0.477	Peroxisome	III
*OF14282*	PbARF11	680	75,645.98	5.99	58.56	67.81	−0.553	Nucleus	V
*OF21754*	PbARF12	787	87,876.70	6.56	52.77	72.35	−0.459	Nucleus	V
*OF09049*	PbARF13	511	56,529.57	8.04	49.50	88.18	−0.221	Chloroplast	IV
*OF05709*	PbARF14	707	77,550.83	7.56	46.17	74.61	−0.350	Nucleus	II
*OF05094*	PbARF15	899	99,896.26	6.06	63.09	78.08	−0.471	Nucleus	III
*OF07141*	PbARF16	705	77,183.54	6.56	50.96	75.80	−0.301	Nucleus	II
*OF20357*	PbARF17	662	73,604.32	6.60	51.51	72.73	−0.410	Peroxisome	IV
*OF20735*	PbARF18	856	95,038.56	5.82	55.64	64.68	−0.620	Nucleus	V
*OF22918*	PbARF19	828	93,545.12	6.11	60.73	71.11	−0.505	Nucleus	III
*OF26137*	PbARF20	1113	122,852.38	6.13	60.47	74.59	−0.518	Nucleus	III
*OF14279*	PbARF21	311	35,145.69	6.40	63.31	71.45	−0.430	Nucleus	V
*OF18244*	PbARF22	815	91,853.67	5.88	60.23	75.24	−0.437	Nucleus	III
*OF15400*	PbARF23	910	100,774.16	6.06	71.90	76.27	−0.449	Nucleus	III
*OF24604*	PbARF24	738	81,011.35	7.35	54.56	70.26	−0.437	Nucleus	IV
*OF01686*	PbARF25	900	99,660.42	6.17	63.19	80.14	−0.383	Nucleus	III

Note: AA: number of amino acids; MW: molecular weight; pI: theoretical isoelectric point; II: instability index; AI: aliphatic index; GRAVY: grand average of hydropathicity.

## Data Availability

Publicly available datasets were analyzed in this study.

## Supplementary Materials

The following supporting information can be downloaded at: https://www.mdpi.com/article/10.3390/ijms26083701/s1.
